# Combined OX40 Agonist and PD-1 Inhibitor Immunotherapy Improves the Efficacy of Vascular Targeted Photodynamic Therapy in a Urothelial Tumor Model

**DOI:** 10.3390/molecules26123744

**Published:** 2021-06-19

**Authors:** Ricardo G. Alvim, Petrina Georgala, Lucas Nogueira, Alexander J. Somma, Karan Nagar, Jasmine Thomas, Laura Alvim, Amelia Riegel, Christopher Hughes, Jie Chen, Augusto B. Reis, Souhil Lebdai, Avigdor Scherz, Steven Zanganeh, Rui Gardner, Kwanghee Kim, Jonathan A. Coleman

**Affiliations:** 1 Urology Service, Department of Surgery, Memorial Sloan Kettering Cancer Center, New York, NY 10065, USA; nogueirl@mskcc.org (L.N.); ajsomma@gmail.com (A.J.S.); nagark@mskcc.org (K.N.); thomasj7@mskcc.org (J.T.); laurapalvim@gmail.com (L.A.); ameliariegel@gmail.com (A.R.); hughesc@mskcc.org (C.H.); chenj4@mskcc.org (J.C.); souhil.lebdai@gmail.com (S.L.); kimk1@mskcc.org (K.K.); 2 Department of Surgery, Federal University of Minas Gerais, Belo Horizonte 30130-100, Brazil; augusto.urologia@gmail.com; 3 Department of Neurosurgery, Memorial Sloan Kettering Cancer Center, New York, NY 10065, USA; 4 Department of Plants and Environmental Sciences, The Weizmann Institute of Science, Rehovot 76100, Israel; avigdor.scherz@weizmann.ac.il; 5 Department of Bioengineering, University of Massachusetts, Dartmouth, MA 02747, USA; steven.zanganeh@gmail.com; 6 Flow Cytometry Core Facility, Memorial Sloan Kettering Cancer Center, New York, NY 10065, USA; gardnerr@mskcc.org

**Keywords:** bladder cancer, tumor ablation, TOOKAD, focal therapy, immunotherapy

## Abstract

Purpose: Vascular targeted photodynamic therapy (VTP) is a nonsurgical tumor ablation approach used to treat early-stage prostate cancer and may also be effective for upper tract urothelial cancer (UTUC) based on preclinical data. Toward increasing response rates to VTP, we evaluated its efficacy in combination with concurrent PD-1 inhibitor/OX40 agonist immunotherapy in a urothelial tumor-bearing model. Experimental design: In mice allografted with MB-49 UTUC cells, we compared the effects of combined VTP with PD-1 inhibitor/OX40 agonist with those of the component treatments on tumor growth, survival, lung metastasis, and antitumor immune responses. Results: The combination of VTP with both PD-1 inhibitor and OX40 agonist inhibited tumor growth and prolonged survival to a greater degree than VTP with either immunotherapeutic individually. These effects result from increased tumor infiltration and intratumoral proliferation of cytotoxic and helper T cells, depletion of Treg cells, and suppression of myeloid-derived suppressor cells. Conclusions: Our findings suggest that VTP synergizes with PD-1 blockade and OX40 agonist to promote strong antitumor immune responses, yielding therapeutic efficacy in an animal model of urothelial cancer.

## 1. Introduction

Urothelial carcinoma (UC) represents the fifth most common malignancy among men in the United States and is most prevalent among men over 60 years old. Between 5% and 10% of primary urothelial cancers originate from the ureter or renal pelvis and are collectively called upper tract urothelial cancers (UTUCs) [1,2].

In patients with low-grade, small UTUC tumors, as well as those in whom functional preservation is especially critical (i.e., those with a single kidney, poor renal function, or bilateral disease), local ablative therapies have emerged as a means of reducing treatment-related morbidity. One such minimally invasive therapy is vascular targeted photodynamic (VTP) therapy, which eradicates cancer cells by targeting the vascular compartment of the tumor while preserving healthy tissue [3,4]. VTP therapy employing WST11 (TOOKAD Soluble, Steba Biotech, Luxembourg) was clinically approved in Europe and Mexico in 2017 for early-stage localized prostate cancer [5,6,7] and has demonstrated efficacy in multiple animal models of UTUC [7,8]. WST-11 is a photosensitizer that is administered intravenously and has a 60 min half-life, avoiding long-term toxicity. Confined laser illumination supplied via optical fibers inserted into the malignant tissue activates the circulating drug locally and induces rapid release of toxic, short-lived free radicals inside tumor blood vessels, leading to vascular occlusion and ultimately tumor necrosis 48 h post-treatment [9].

Though VTP can eradicate UTUC tumors, the cure rate in the standard mouse model is only approximately 30%. To increase its efficacy, VTP has been combined with immune checkpoint inhibitors based on the observation that it induces antitumor immune responses through a prominent and rapid inflammatory reaction [10]. The synergy of VTP with checkpoint inhibitors such as anti-PD-1 and anti-CTLA-4 has been demonstrated in animal studies, in which the complete response rate is greater than 50% [7,11].

To maximize the clinical benefit of VTP, adding another immunotherapeutic agent is a promising approach. Combining immunomodulatory drugs with immune checkpoint inhibitors has received considerable attention as a means of increasing the benefit of the latter, which can induce potent clinical and immunologic responses in a minority of patients [12]. An attractive immunomodulatory target is the T cell costimulatory receptor OX40 (CD134), a member of the tumor necrosis factor receptor superfamily (TNFRSF) that acts upon T cell signaling pathways that promote survival, proliferation, and cytokine production [13]. Preclinical studies have shown that OX40 agonist antibodies can enhance antitumor immunity through T cell expansion, leading to improved progression-free survival [14,15,16,17]. OX40 agonism has been shown to augment the efficacy of checkpoint inhibitors in models of multiple cancers [18,19,20].

In addition, high-dose radiation has also been shown to transform the immunosuppressive tumor microenvironment, resulting in an intense CD8^+^ T-cell tumor infiltrate and improving the tumor ablation effect. This transformation is dependent on antigen cross-presenting CD8^+^ dendritic cells, secretion of IFNγ, and CD4^+^ T cells expressing CD40L, opening the concept of OX40 plus ablation therapy (VTP) can increase this effect [21].

We postulated that combining VTP with PD-1 blockade and OX40 agonistic immunotherapy would enhance the therapeutic efficacy of VTP in urothelial cancer by inducing long-lasting antitumor immunity.

## 2. Results

### 2.1. Combination of PD-1 Blockade and OX40 Agonism with VTP Therapy Suppresses Tumor Growth and Prolongs Survival

**Figure 1:**
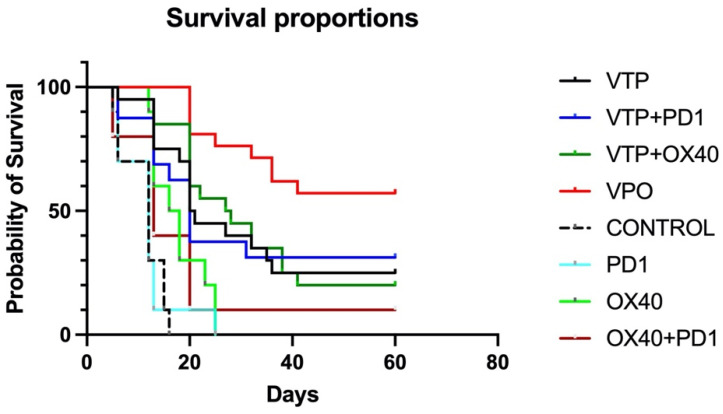
Combined treatment with OX40 agonist and PD1 inhibitor antibodies following VTP therapy suppresses primary tumor growth and improves survival. Percent survival of mice plotted by Kaplan-Meier methods (*p* < 0.0001).

To evaluate the antitumor therapeutic effect of combining VTP treatment with the combination immunotherapy, MB-49 UTUC allograft-bearing mice were treated with VTP on day 15 following tumor implantation and injected the following day with OX40 agonist and PD-1 inhibitor antibodies, as described above. Five out of 80 mice (6.25%) treated with VTP died in the first 72 h, related to intense inflammatory reaction from the VTP treatment, and were excluded from the study. For comparison, other mice were treated with one of the component therapies or a dual combination. Compared to controls, the immunomodulatory agent groups (OX40 agonist only, PD-1 inhibitor only and dual combination) significantly delay tumor growth (*p* < 0.0001), however no difference was observed between the different schemes (*p* = 0.59; Supplementary Figure S1). Treatment of primary MB-49 tumors with VTP reduced tumor growth compared with control and immunomodulatory agent-treated animals. Fourteen days after VTP ablation, 42% (8 out 19) of VTP only-treated mice did not display tumors and the average volume was 442 mm^3^, while mice in the latter treatment groups developed tumors with almost double size (average volume > 800 mm^3^, *p* = 0.04, Supplementary Figure S1). While single-agent treatment with either PD-1 inhibitor or OX-40 agonist had limited effect, one mouse (10%) treated with both drugs had a complete tumor regression and survived until the end of study (Figure 1). Combined VTP plus PD-1 inhibitor plus OX40 agonist therapy (VTP+OX40+PD-1) dramatically reduced tumor burden compared with mice treated with either VTP alone or VTP plus a single drug (Supplementary Figures S1 and S2, *p* < 0.0001). VTP+OX40+PD-1 treatment significantly increased survival to 60% at 60 days compared with 25% in mice treated with VTP alone, 31.25% in those given VTP plus PD-1 inhibitor, 20% for VTP plus OX40 agonist (*p* < 0.0001; Figure 1). All non-VTP-treated animals, but one exception in OX40 + PD1 group, died around 30 days after VTP treatment (Figure 1).

### 2.2. Combination Therapy with VTP Followed by PD-1 Inhibitor and OX40 Agonist Treatment Enhances T Cell Infiltration into Urothelial Tumor Tissue

**Figure 2:**
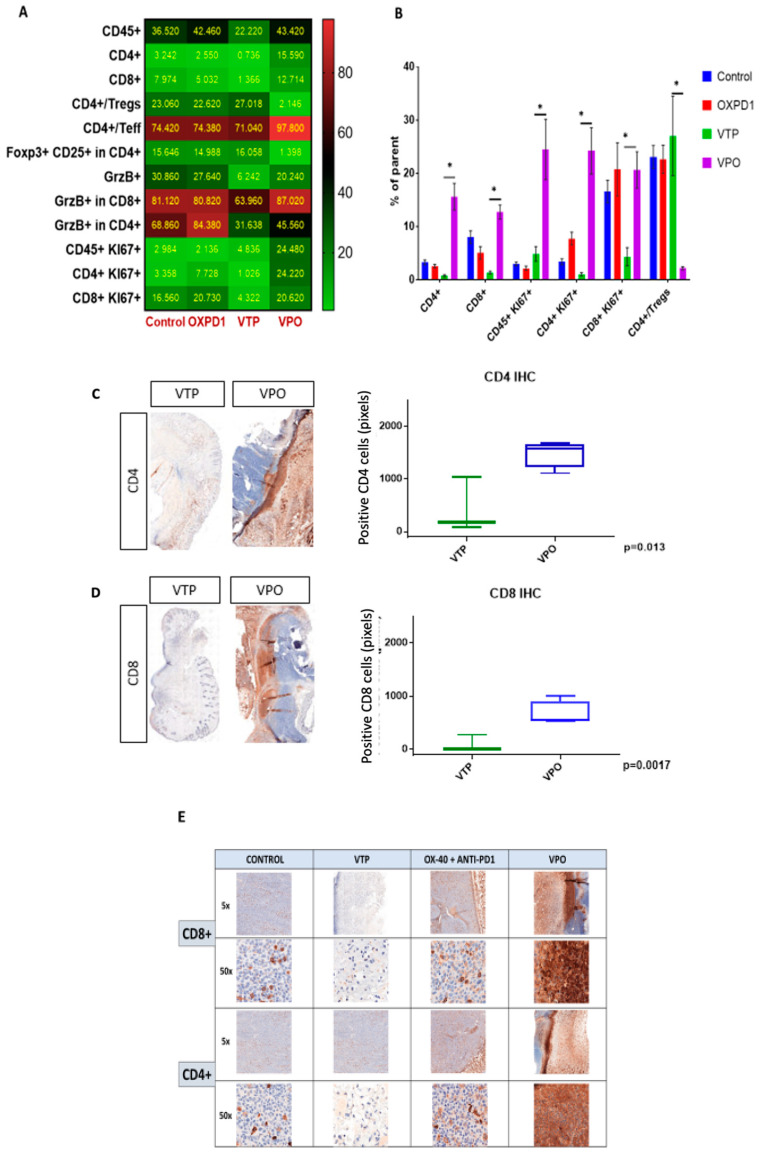
Adding PD-1 inhibitor and OX40 agonist to VTP therapy increases T cell infiltration and activation into MB-49 UTUC allografts at 7 days post-treatment and increases CD4^+^ and CD8^+^ T cell infiltration into MB-49 UTUC allografted tumors. (**A**) Heat map showing the mean frequency of each cell population as measured by flow cytometry in control and treated tumors. Each box represents the mean percentage of positive cells among the parent cell population, i.e., CD45^+^ among live cells, CD4^+^ or CD8^+^ cells among CD45^+^ cells, Foxp3^+^ Tregs or Teff among CD4^+^ cells, CD25^+^/Foxp3^+^ Tregs among CD4^+^ cells, GrzB^+^ among CD45^+^ cells, GrzB^+^ cells among CD8^+^ or CD4^+^ cells, Ki67^+^ cells among CD45^+^ cells, and Ki67^+^ among CD8^+^ or CD4^+^ cells. (**B**) Frequency of positive cells among the parent cell population. Graph bars indicate mean ± SEM. * *p* < 0.01. OXPD1, OX40 agonist plus PD1 inhibitor; VTP+OX40+PD-1, VTP plus PD1 inhibitor and OX40 agonist. (**C**) A representative IHC of CD4^+^ T lymphocytes in VTP+OX40+PD-1-treated tumors compared with VTP followed by the signal intensity comparison showing a more significant CD4^+^ staining in VTP+OX40+PD-1 group compared to VTP alone (*p* = 0.013). (**D**) A representative IHC of CD8^+^ T lymphocytes in VTP+OX40+PD-1-treated tumors compared with VTP followed by the signal intensity comparison showing a more significant CD8^+^ staining in VTP+OX40+PD-1 group compared to VTP alone (*p* = 0.0017). (**E**) A representative microscopy IHC illustration of CD8^+^ T lymphocytes and CD4^+^ T lymphocytes difference between control, VTP, OX40 plus PD1 and VTP+OX40+PD-1 groups. In both staining, VTP+OX40+PD-1 presented a clear high intensity signal.

As the goal of cancer immunotherapy is to elicit strong, durable tumor-specific T cell responses capable of eliminating malignant tumors, we compared T cell recruitment among treatment groups at day 7 after VTP treatment by multi-color flow cytometry. As seen in controls, these tumors normally contain few CD8^+^ and CD4^+^ T cells and abundant CD4^+^ Foxp3^+^CD25^+^ Treg cells (Figure 2A,B). After VTP treatment, CD4^+^ and CD8^+^ were decreased (Figure 2C,D). In accordance with the observed therapeutic response following combined VTP with OX40 agonist/PD-1 inhibitor treatment (Figure 2), this combination led to a striking increase in intratumoral CD8^+^ and CD4^+^ T cells compared to VTP alone, at day 7 post-treatment (Figure 2). Immunohistochemistry at day 7 after treatment confirmed the presence of high numbers of CD8^+^ and CD4^+^ T lymphocytes in VTP+OX40+PD-1-treated tumors compared with VTP alone group (Figure 2C,E). Quantification of CD4 and CD8 signal intensity in tumors indicated that this increase was significant (*p* < 0.01; Figure 2C,D). Careful examination of tumor sections revealed that most infiltrating T cells were recruited close to the tumor boundary (Figure 2C,E).

**Figure 3:**
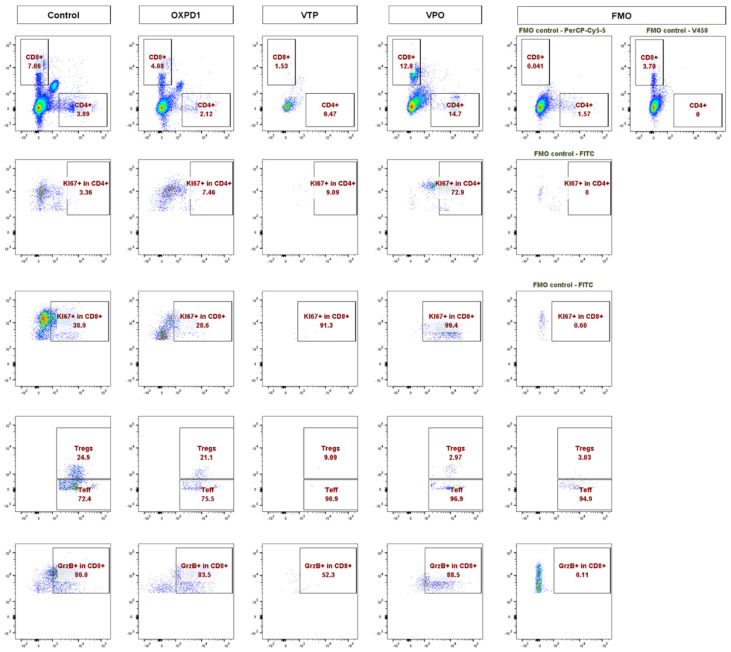
Adding PD-1 Inhibitor and OX40 agonist to VTP therapy increases T cell infiltration and activation compared to VTP alone, into MB-49 UTUC allografts at 7 days post-treatment. Representative flow cytometry plots showing the percentage of CD4^+^ and CD8^+^ cells among CD45^+^ cells (top row), Ki67^+^ in CD4^+^ (second row), Ki67^+^ in CD8^+^ (third row), Foxp3^+^ Tregs and Foxp3^−^ Teff in CD4^+^ (fourth row), and GrzB^+^ in CD8^+^ T cells (bottom row) in control and treated tumors analyzed 7 days after treatment. A fluorescent minus one (FMO) sample was included for each marker as a negative control for gating.

In light of these data, we further characterized the CD8^+^ and CD4^+^ T cell phenotypes. We found that both CD8^+^ and CD4^+^ T cell populations were more proliferative at day 7, the latter by 5- to 10-fold, in VTP+OX40+PD-1-treated mice compared with VTP alone, as evidenced by abundant co-expression of the proliferation marker Ki67 (Figure 2A,B). We also observed significantly increased Ki67 expression within the CD45^+^ population in VTP+OX40+PD-1-treated tumors (*p* < 0.01, Figure 2A,B and Figure 3). Such markers of cellular proliferation can be used as surrogates for T cell activation, given that activation is required for proliferation. We then evaluated granzyme B expression, which is associated with the cytotoxic activity of CD8^+^ T cells. Granzyme B expression was upregulated in the CD8^+^ population of VTP+OX40+PD-1-treated tumors compared with VTP-treated tumors (though similar to that in control and immunomodulator-treated tumors; Figure 2B).

Overall, the increase in CD8^+^ and CD4^+^ T lymphocyte infiltration and proliferation, and activation of cytotoxic CD8^+^ T cells associated with combining OX40 agonist and PD-1 inhibitor with VTP indicates that this therapeutic approach promotes antitumor immunity.

### 2.3. Combined PD-1 Inhibitor/OX40 Agonist Immunotherapy Diminishes Immunosuppression in VTP-Treated Tumors

**Figure 4:**
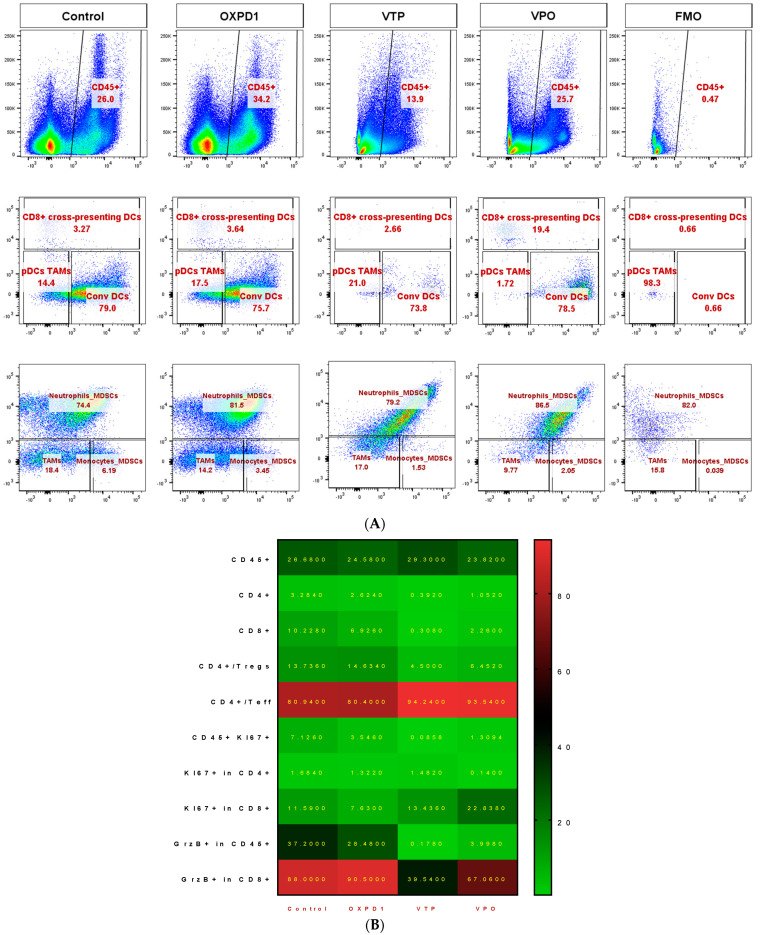

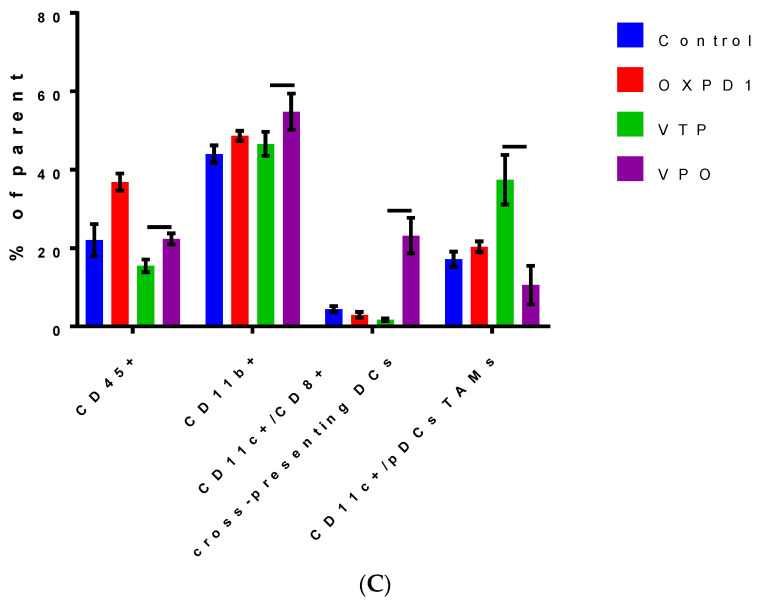
Combined PD-1 inhibitor/OX40 agonist therapy alters the frequency of specific myeloid cell subsets in VTP-treated tumors by day 7 post-treatment. (**A**) Representative flow cytometry plots identifying myeloid cell subsets within viable single cells. (**B**) Heat map summarizing the findings of flow cytometry analyses of tumor samples at day 7 after treatment. Values in boxes represent the mean percentage of each cell subpopulation (highlighted by row) in each experimental condition (highlighted by column). (**C**) Plot of flow cytometry data shown as the percentage of positive events among parent gated events. DCs, dendritic cells; pDCs, plasmacytoid dendritic cells; Conv DCs, conventional dendritic cells; TAMs, tumor-associated macrophages; MDSCs, myeloid-derived suppressor cells. Graph bars indicate mean ± SEM. OXPD1, OX40 agonist plus PD-1 inhibitor; VTP+OX40+PD-1, VTP plus PD-1 inhibitor and OX40 agonist.

We evaluated the efficacy of combined inhibition of the immune checkpoint PD-1 and activation of the costimulatory OX40 in blocking immunosuppression in MB-49 UTUC allografts by quantifying regulatory cell populations including regulatory T cells (Tregs), myeloid derived suppressor cells (MDSCs) and tumor-associated macrophages (TAMs). The addition of PD-1 blockade and OX40 activation to VTP reduced Treg numbers in tumors at day 7 post-VTP (Figure 2A,B); VTP+OX40+PD-1-treated tumors contained drastically fewer Tregs compared with controls and those treated with VTP or both immunomodulators. Furthermore, we found that TAMs were significantly reduced in VTP+OX40+PD-1-treated compared with VTP-treated tumors, whereas CD8^+^CD11c^+^ cross-presenting DCs were significantly elevated in VTP+OX40+PD-1-treated tumors compared to VTP alone or immunomodulators (OX40 agonist plus PD-1 inhibitor) without VTP (Figure 4A–C; *p* < 0.01). Overall, these findings suggest that combining PD-1 inhibition and OX40 activation with VTP reduces numbers of immune-suppressive cells. As depletion of Tregs has been correlated with enhanced antitumor responses, this agrees with our findings of increased intratumoral T cell accumulation and survival in VTP+OX40+PD-1-treated mice.

We also performed studies using mice bearing 4T1 breast tumor cells. This is an aggressive breast cancer model with fast metastatic development, even when the primary tumor is still relatively small (<80 mm^3^). However, at this time, many animals already had metastatic disease, and they died regardless of the primary tumor treated by VTP. In this validation model, outcomes were improved in the VTP+PD-1+OX40 group compared to the others, but the overall survival was not. In this study, no animal was sacrificed because of the primary tumor growth (volume > 2000 mm^3^). Indeed, they died even after complete ablation of the primary tumor, showing that our timepoint for VTP treatment at two weeks was too late for disease control (supplementary Figure S3).

## 3. Materials and Methods

### 3.1. Cell Culture and Tumor Model

Luciferase-labelled MB-49 cells were obtained from the American Type Culture Collection (ATCC; Manassas, VA, USA) and cultured in RPMI medium supplemented with 10% fetal calf serum (Life Technologies/Thermo Fisher Scientific, Waltham, MA, USA). Seven- to eight-week-old male C57BL/6J mice were purchased from the National Cancer Institute (Frederick National Laboratory for Cancer Research, Frederick, MD, USA) and housed and treated according to a protocol approved by the MSKCC Institutional Animal Care and Use Committee (IACUC). Before tumor implantation, mice were anesthetized with inhaled isoflurane, meloxicam (2 mg/kg), and buprenorphine (0.5 mg/kg), and the hair and skin overlying the right flank was sterilized using a povidone-iodine and ethyl alcohol solution. MB-49 cells (50,000) were implanted in the right flank.

Fourteen days after tumor implantation, mice were assessed for tumor formation using noninvasive bioluminescence imaging. Animals were retro-orbitally injected with D-luciferin (200 mg/kg) 5 min before undergoing a 30-s scan using an IVIS Spectrum Optical Imaging System (Xenogen, Alameda, CA, USA). Regions of interest from displayed images were drawn based on signal intensity and quantified using Living Image software version 2.60. Specific signal was calculated as the ratio of bioluminescent signal in the region of interest to the bioluminescent signal in a background region containing no tumors. When tumor volume reached approximately 100 mm^3^, animals were randomly assigned to the following cohorts for future experiments: controls (*n* = 10), PD-1 inhibitor (*n* = 10), OX40 agonist (*n* = 10), PD-1 inhibitor plus OX40 agonist (*n* = 10), VTP (*n* = 20), VTP plus OX40 agonist (*n* = 20), VTP plus PD-1 inhibitor (*n* = 20) and VTP plus OX40 agonist and PD-1 inhibitor (*n* = 20) (Table 1). For survival and tumor growth comparison, the experiment was made in two steps, and the results were put together for statistical analysis.

### 3.2. WST11-VTP and Immunomodulatory Treatment and Tumor Follow-Up

Fifteen days after tumor implantation, tumor-bearing mice were anesthetized by intraperitoneal (i.p.) injection of a mixture of ketamine (150 mg/kg body weight) and xylazine (10 mg/kg) plus inhaled isoflurane administered via a tight-fitting nose cone. Following retro-orbital injection of WST11 (9 mg/kg bodyweight), tumors were illuminated with 753 nm light delivered via diode laser with a 600 μm end-fire fiber (Biolitec, East Longmeadow, MA, USA) for 10 min at 150 mW/cm^2^. In the PD-1 inhibitor subgroups, InVivoPlus antimouse PD-1 CD279-Clone: 29F.1A12 (250 mg/kg, Bioxcell, West Lebanon, NH, USA) was injected intraperitoneally (IP) on days 1, 4, 7, 10, 13 and 16 after VTP. In the OX40 agonist subgroups, InVivoPlus antimouse OX40 CD134-Clone: OX-86 (500 mg/kg, Bioxcell, West Lebanon, NH, USA) was injected IP in a single dose on day 1 after VTP.

At least once a week until 60 days after VTP treatment, tumor volume was measured, the animals’ health was checked, and IVIS spectrum imaging was performed to monitor local tumor and lung metastasis. When tumors exceeded 2000 mm^3^ or lung metastasis developed, mice were euthanized via 100% carbon dioxide at 5 PSI for a minimum of 3 min, at a displacement rate of 30% chamber volume/minute in a cage or euthanasia chamber. These euthanasia practices are in accordance with our institution’s Research Animal Resource Center’s Recommended Methods of Euthanasia for laboratory Animals and the American Veterinary Medical Association Guidelines for the Euthanasia of Animals. For statistical analysis, we compared tumor volume until 30 days after tumor injection (Day 14 pos-VTP), when tumor volume in the control group reached the protocol limit (2000 mm^3^).

### 3.3. Immunohistochemistry

Immunohistochemistry (IHC) was performed for CD3, CD4, CD8, and OX40 (CD134). IHC was performed on CD3, CD4, and CD8 using a Leica Bond RX automated strainer with bond reagents (Leica Biosystems, Buffalo Grove, IL, USA), including a polymer detection system (DS9800, Novocastra Bond Polymer Refine Detection, Leica Biosystems, Buffalo Grove, IL, USA). The chromogen was 3,3 diaminobenzidine tetrachloride (DAB), and sections were counterstained with hematoxylin. IHC was performed manually on OX40 (CD134) using an avidin-biotin detection system (Vectastain Elite ABC HRP Kit, Vector Laboratories, Burlingame, CA, USA). The chromogen was 3,3 diaminobenzidine tetrachloride (DAB), and sections were counterstained with hematoxylin. Details for each marker are shown in the Supplementary Table S1.

Slides were digitally scanned using a Pannoramic Flash 250 digital scanner (3DHistech, Budapest, Hungary) and a Zeiss 20x/0.8NA objective. Relevant tissue regions were denoted and analyzed using the Pannoramic Viewer (3DHistech, Budapest, Hungary). CD3, CD4, CD8, and OX40 staining was quantified within tumors by manual counting of positive cells within defined regions of equal area.

### 3.4. Flow Cytometry

Single-cell suspensions were prepared from spleen, lymph nodes, or dissociated tumors after mechanical dissociation and passage through a 40 µm nylon filter. For all flow cytometry experiments, single-cell suspensions were maintained in flow cytometry buffer (PBS, 2% FBS) and blocked with rat antimouse CD16/32 for 15 min at 4 °C before incubating with the appropriate antibody cocktails in the dark for 30 min at 4 °C. Intracellular staining was performed using Cytofix/Cytoperm reagents (BD Biosciences, San Jose, Cam USA, Catalog No 554714) according to manufacturer’s directions. Antibodies used for the myeloid panel include CD45.2-FITC (BD 553772), CD11c-PE (BD 557401), CD8a-PE-Texas Red (Life Technologies MCD0817), Ly6G-PerCP-Cy5.5 (BD 560602), Ly6C-PE-Cy7 (5236982), MHC II-eFluor450 (eBioscience 48-5321-82), CD86-APC (BD 558703), CD11b-APC/eFluor 780 (eBioscience 47-0112-82), and. Antibodies used for the T cell activation panel include Ki67-FITC (eBioscience 11-5698-82), CD62L-PE (BD 553151), GrzB-PE/Dazzle 594 (Biolegend 372216), CD8a-PerCP-Cy5.5 (BD 551162), CD44-PE/Cy7 (Biolegend 103030), CD4-V450 (BD 560468), Foxp3-APC (eBioscience 17-5773-82), CD45.2-Alexa Fluor700 (eBioscience 56-0454-82), CD25-APC/Cy7 (BD 557658). Both panels also included eBioscience Fixable viability dye-eFluor506 (ThermoFisher, New York, NY, USA, 65-0866-14). Cells were quantified using an LSRFortessa analyzer (BD Biosciences, San Jose, CA) equipped with 350 nm, 405 nm, 488 nm, 561 nm, and 640 nm excitation lasers. Data was collected using BD FACS Diva software (BD Biosciences) and analyzed using FlowJo software (Tree Star, Ashland, OR, USA). During acquisition, a live CD45^+^ gate was set according to scatter parameters, live/dead and CD45 staining. A minimum of 1–4 ×10^5^ live CD45+ cells were acquired per sample. Compensation was set using UltraComp beads (ThermoFisher Scientific, New York, NY, USA, Cat No 01-2222-41) stained with individual fluorochromes and cells stained with live/dead dye. Compensation matrices were calculated and applied using FlowJo software. Unstained cells were acquired for each tissue type analyzed and fluorescence minus one (FMO) controls were used for gating to distinguish positively from negatively stained cell populations.

### 3.5. Statistical Analyses

GraphPad Prism was used for all statistical analyses. The Kruskal–Wallis test was used to assess differences among all groups and t-test was used for pairwise comparison of means between groups. A Kaplan–Meier curve was built for survival comparison. *p* < 0.05 was defined as significant.

## 4. Discussion

Photodynamic therapy is a minimally invasive tissue ablation modality in which a photosensitizing substance is activated through exposure to laser light radiation delivered at a specific wavelength. In the presence of oxygen, this triggers a photochemical reaction that generates oxidant species (radicals, singlet oxygen, triplet species), leading to targeted tissue destruction through direct cytotoxicity, vascular shutdown, and activation of an immune response. Our study highlights combination immunotherapy as a potential means to enhance the efficacy of VTP treatment. We show that combining VTP treatment with an immune checkpoint inhibitor and an immune costimulatory receptor agonist reduces tumor burden and enhance survival to a greater degree than VTP or the immunomodulators alone in a mouse model of urothelial cancer. We further demonstrate that these antitumor effects occur mainly in a T cell-dependent manner, namely enrichment of cytotoxic and helper T cells in the tumor area and depletion of Treg cells, and that the increase in intratumoral T cells results in part from enhanced proliferation. The triple combination also acts via a T-cell independent mechanism to promote antitumor immunity, as it reduces numbers of intratumoral MDSCs.

The efficacy of this combination fits well with prior evidence showing that urothelial cancers are highly immunogenic malignancies. This immunogenicity has been known for decades, since the discovery that bacillus Calmette–Guerin slows the growth of bladder cancer by invoking immunological activity [22]. Similarly, immune checkpoint inhibitors have proven beneficial as salvage therapy in metastatic urothelial cancer [23,24], in accordance with its high mutation frequency [25], which presumably leads to greater neoantigen burden [26], known to correlate with checkpoint inhibitor efficacy [27]. Immune responses are also correlated with tumor aggressiveness even in the absence of immunotherapies, as numbers of CD8^+^ T cells are recently described as predictive of survival in muscle-invasive and advanced urothelial carcinoma [28,29].

The benefit of combining VTP with PD-1/PD-L1 blockade and OX40 agonism (VTP+OX40+PD-1 treatment) may extend to other cancers, especially those in which two of the component therapies have been shown to synergize in animal models. These include lung and kidney cancer, in which O’Shaughnessy et al. showed recently that adding anti-PD-1 or anti-PD-L1 to VTP reduces metastasis and improves survival [11]. As Preise et al. showed that VTP induces antitumor immunity that is cross-reactive between colon and breast cancers, these might also respond to the combination [10].

Our study extends the range of therapies with which OX40 agonism has been shown to synergize. In addition to immune checkpoint inhibitors, OX40-stimulating antibodies have been shown to augment the effectiveness of several treatments that indirectly stimulate immune responses, including dasatinib (in a c-KIT mutant P815 mastocytoma tumor model [30], dabrafenib and trametinib (in BRAF^V600E^-mutant melanoma) [31], and radiotherapy [32,33,34].

We found that combination VTP plus PD-1 inhibition plus OX40 agonist (VTP+OX40+PD-1) treatment inhibits immune suppression by reducing the intratumor prevalence of regulatory T cells (Tregs), tumor-associated macrophages (TAMs) and, especially, myeloid-derived suppressor cells (MDSCs). MDSCs have been the focus of intense research in recent years; high numbers of these immunosuppressive cells have been correlated with tumor aggressiveness and poor prognosis, including in urothelial cancer [35,36]. Our laboratory has recently shown that targeting MDSCs using anti-CSF1R in combination with VTP therapy not only decreased the numbers of intratumoral MDSCs and TAMs, but also increased CD8^+^ T cell infiltration, decreasing tumor growth and improving overall survival in a prostate cancer model [37]. Thus, the reduction of TAMs and MDSCs by the VTP+OX40+PD-1 combination may have been a major contributor to its efficacy.

The strong T cell recruitment induced by VTP+OX40+PD-1 treatment—greater than that following treatment with immunomodulators in the absence of VTP—is an important finding of our study. Thus, adding VTP could overcome a limitation of these immunotherapies, which are generally more effective in mice bearing small tumors. This synergistic effect on T cells confirms Aspeslagh et al.’s postulation that therapies that increase antigen release, a known effect of VTP [38], could improve the potency of OX40 stimulation [13]. Indeed, VTP+OX40+PD-1 provided greater local tumor control compared with PD-1 inhibition and OX40 agonism, including complete remission in 66% of the animals. We speculate that the VTP-induced acute inflammatory response acts as an immune booster, releasing antigens to which immunotherapy-stimulated and deinhibited T cells react.

## 5. Conclusions

In summary, we provide evidence that OX40 agonism plus PD-1 inhibitor immunotherapy is a valuable combinatorial approach that improves the efficacy of VTP in a urothelial cancer model. The results presented here support the concurrent manipulation of these two immune pathways to maximize the ability of VTP treatment to provide local tumor control and improve survival. This study, along with prior findings, indicates that the addition of immunotherapy to VTP treatment is an effective therapeutic strategy for reducing immunosuppression within tumors and ultimately enhancing antitumor immunity.

## Figures and Tables

**Table 1:** Therapeutic schemes received by each group.

GROUPS	PD-1	OX40	PD-1+OX40	VTP	VTP+PD-1	VTP+OX40	VTP+OX40+PD-1	CONTROL
INTERVENTION								
Antibody anti OX-40		**X**	**X**			**X**	**X**	
Antibody anti-PD-1	**X**		**X**		**X**		**X**	
VTP *				**X**	**X**	**X**	**X**	
NO INTERVENTION								**X**

* Vascular Target Photodynamic therapy (150J).

## Supplementary Materials

The following are available online: Table S1: Immunohistochemistry (IHC) for CD3, CD4, CD8. Figure S1: Growth patterns individed by VTP and no VTP groups. Figure S2: Metastatic signaling by IVIS following VTP Figure S3: Survival outcomes after VTP in the 4T1 model.

## Sample Availability

Samples are not available from authors.
