# Supplementary material for: Combined OX40 Agonist and PD-1 Inhibitor Immunotherapy Improves the Efficacy of Vascular Targeted Photodynamic Therapy in a Urothelial Tumor Model
Source: Molecules. 2021 Jun 19;26(12):3744. doi: 10.3390/molecules26123744 (PMC8234268; doi:10.3390/molecules26123744)
Supplement: Supplementary file 1 [file molecules-26-03744-s001.zip › molecules-1193741-supplementary.pdf]

Supplementary Materials

Table S1. Immunohistochemistry (IHC) for CD3, CD4, CD8.

Marker	Epitope Retrieval	Primary Antibody Source, Catalog Number	Primary Antibody Concentration	Secondary Antibody Source, Catalog Number	Secondary Antibody Concentration
CD3	Heat induced, pH 6.0	Abcam, ab135372	1:250	Leica Biosystems, DS9800 kit, reagent #3	Used at concentration provided by vendor
CD4	Heat induced, pH 9.0	eBioscience, 14-9766-82	1:250	Vector Laboratories, BA-4001	1:100
CD8	Heat induced, pH 6.0	eBioscience, 14-0808	1:1000	Vector Laboratories, BA-4001	1:100
OX40 -(CD134)	Heat induced, pH 6.0	Abcam, ab203220	1:500	Vector Laboratories, BA-1000	1:500

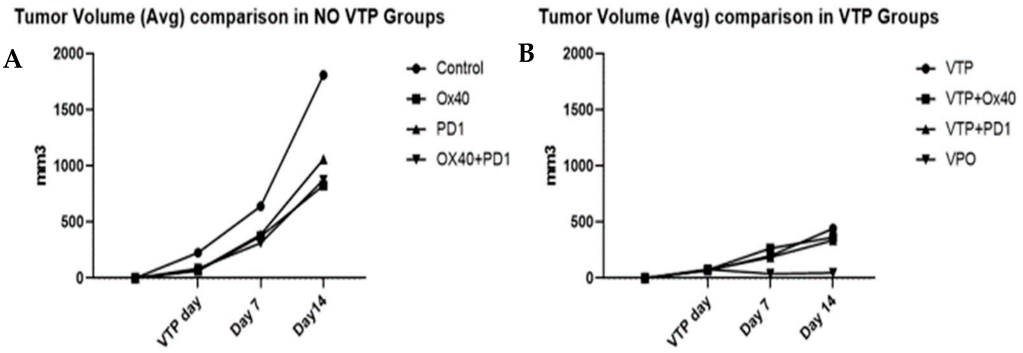

Figure S1. Combined treatment with OX40 agonist and PD1 inhibitor antibodies following VTP therapy suppresses primary tumor growth and improves survival. Mean tumor volumes per group (with Standard deviation). (**A**) Groups without VTP treatment and (**B**) all groups treated with VTP. Both graphs showing significant local tumor control with VPO combination ($p < 0.0001$). VTP, vascular-targeted photodynamic therapy; VPO, vascular-target photodynamic therapy combined with OX-40 and PD-1. $n = 10$ for all cohorts except control, OX40, and PD-1, for which $n = 5$.

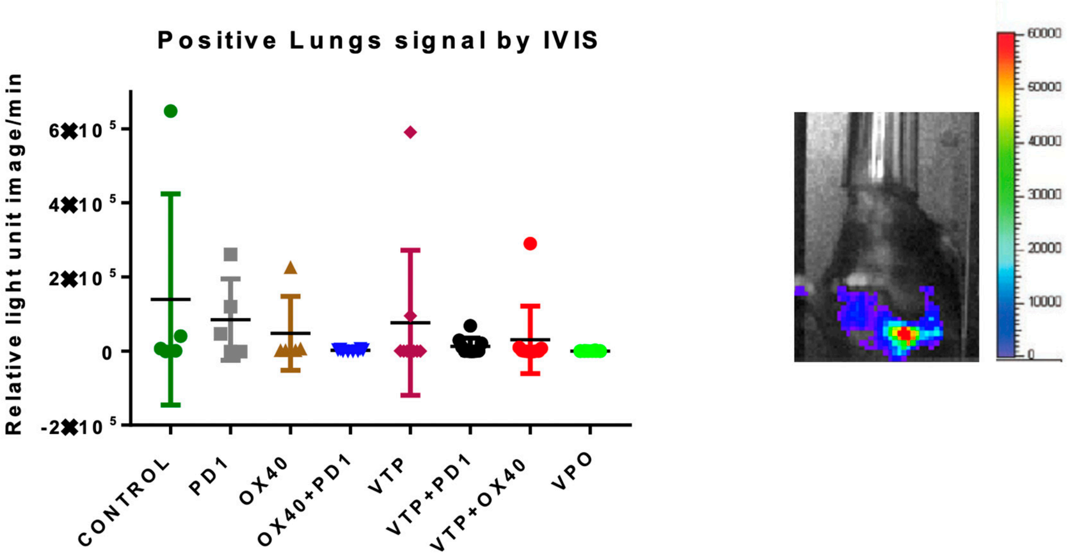

Figure S2. Combined treatment with OX40 agonist and PD1 inhibitor antibodies following VTP therapy suppresses primary tumor metastasis and improves survival.

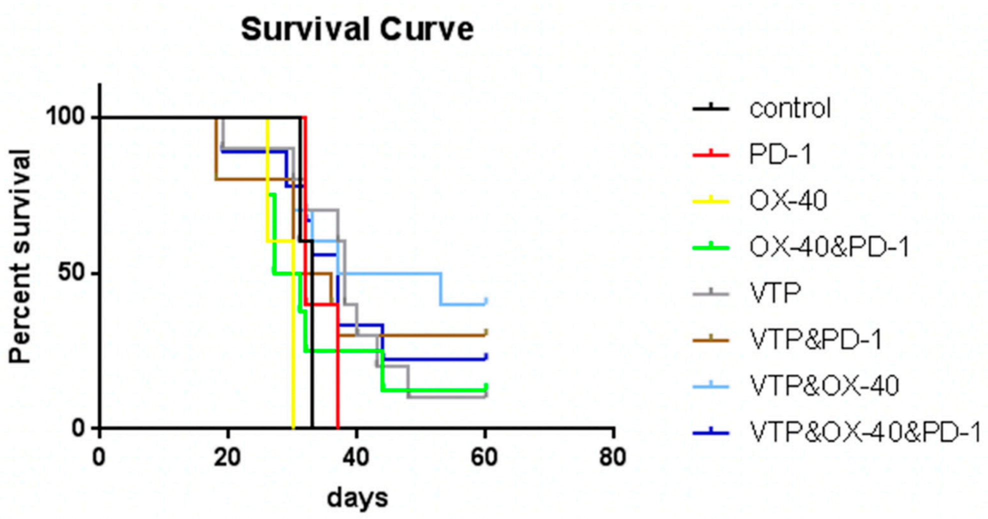

Figure S3. Combined treatment with OX40 agonist and PD1 inhibitor antibodies following VTP therapy suppresses primary tumor growth and improves survival in the 4T1 model.
